# Remote Monitoring for Implantable Defibrillators: A Nationwide Survey in Italy

**DOI:** 10.2196/ijmr.2824

**Published:** 2013-09-20

**Authors:** Mario Luzi, Antonio De Simone, Loira Leoni, Claudia Amellone, Ennio Pisanò, Stefano Favale, Massimo Iacoviello, Raffaele Luise, Maria Grazia Bongiorni, Giuseppe Stabile, Vincenzo La Rocca, Franco Folino, Alessandro Capucci, Antonio D'Onofrio, Francesco Accardi, Sergio Valsecchi, Gianfranco Buia

**Affiliations:** ^1^Azienda Ospedaliero Universitaria Ospedali RiunitiCardiology ClinicAnconaItaly; ^2^Clinica San MicheleMaddaloniItaly; ^3^Azienda Ospedaliera Universitaria di PadovaPaduaItaly; ^4^Ospedale Civile di CiriéCiriéItaly; ^5^Vito Fazzi HospitalLecceItaly; ^6^University of BariBariItaly; ^7^Ospedale San SalvatoreCoppitoItaly; ^8^University Hospital of PisaPisaItaly; ^9^Clinica MediterraneaNaplesItaly; ^10^Azienda Ospedaliera dei Colli – MonaldiNaplesItaly; ^11^Boston ScientificMilanItaly

**Keywords:** implantable defibrillator, remote monitoring, follow-up

## Abstract

**Background:**

Remote monitoring (RM) permits home interrogation of implantable cardioverter defibrillator (ICD) and provides an alternative option to frequent in-person visits.

**Objective:**

The Italia-RM survey aimed to investigate the current practice of ICD follow-up in Italy and to evaluate the adoption and routine use of RM.

**Methods:**

An ad hoc questionnaire on RM adoption and resource use during in-clinic and remote follow-up sessions was completed in 206 Italian implanting centers.

**Results:**

The frequency of routine in-clinic ICD visits was 2 per year in 158/206 (76.7%) centers, 3 per year in 37/206 (18.0%) centers, and 4 per year in 10/206 (4.9%) centers. Follow-up examinations were performed by a cardiologist in 203/206 (98.5%) centers, and by more than one health care worker in 184/206 (89.3%) centers. There were 137/206 (66.5%) responding centers that had already adopted an RM system, the proportion of ICD patients remotely monitored being 15% for single- and dual-chamber ICD and 20% for cardiac resynchronization therapy ICD. Remote ICD interrogations were scheduled every 3 months, and were performed by a cardiologist in 124/137 (90.5%) centers. After the adoption of RM, the mean time between in-clinic visits increased from 5 (SD 1) to 8 (SD 3) months (*P*<.001).

**Conclusions:**

In current clinical practice, in-clinic ICD follow-up visits consume a large amount of health care resources. The results of this survey show that RM has only partially been adopted in Italy and, although many centers have begun to implement RM in their clinical practice, the majority of their patients continue to be routinely followed-up by means of in-clinic visits.

## Introduction

### Remote Monitoring

Remote monitoring (RM) has been developed in order to handle the increasing number of patients with implantable cardiac devices, and who therefore require follow-up visits. Indeed, follow-up visits of implantable cardiac devices are the most frequent activities performed at arrhythmia services [1], and place a great burden on health care providers [2].

RM systems include a patient monitor that, using radiofrequency telemetry, allows data transmission without patient intervention. The patient’s information is sent to a secure network server via the telephone connection. The clinical staff can review device information on a secure Internet-accessible website. These systems provide full device interrogation, monitoring for arrhythmias, and surveillance of device performance from the patient’s home. Moreover, RM systems can alert the physician via phone or email, in the case of programmable parameters, about clinical or device issues. RM has many potential benefits, both for the patient and for the follow-up center. Indeed, it was shown to detect events more quickly and more frequently [3,4], specifically facilitating the early detection of technical issues and clinical anomalies [5], and thus to decrease the time to a clinical decision [6], reduce urgent in-clinic visits [7,8], and mortality [9].

RM systems are currently available for almost all makes of implantable cardioverter defibrillator (ICD) and have been operational in Europe for about 10 years.

### Aim

To date, only few and contradictory data were presented on the actual adoption of RM in routine clinical practice in Italy and Europe [10,11]. The aim of this survey was to investigate the current practice of ICD follow-up in Italy and to evaluate the adoption and routine use of RM.

## Methods

### The Questionnaire

There were 206 Italian centers implanting ICDs that replied to an *ad hoc* questionnaire sent in July 2012.

**Figure 1:**
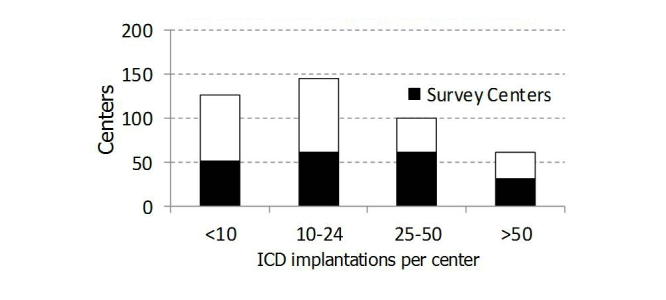
Volume of de novo implantations in the 432 Italian ICD implanting centers and the 206 Italia-RM survey centers in 2011 (published data from the national AIAC Registry).

A complete list of participating centers is reported in Multimedia Appendix 1. The participating centers constituted a representative sample (206/432, 47.7%) of the 432 Italian ICD implanting centers listed in the 2011 edition of the Italian ICD Registry of Italian Society of Arrhythmology and Pacing (AIAC) [12], which includes almost all implanting centers in Italy. According to published data from the AIAC Registry, the survey centers performed 5534 (61.50%) of the 8998 *de novo* ICD implantation procedures carried out in 2011 in Italy. Figure 1 shows the replies to this survey came from centers with a wide range of annual ICD implantation volumes.

The centers were asked to describe their practice of ICD follow-up. Specifically, they reported on the actions performed during routine device follow-up, the time required for follow-up examinations, the involvement of health care personnel, the interval between scheduled follow-up visits, and their use of RM. The complete list of survey questions is listed below.

Routine ICD follow-up:

- Number of ICD patients in follow-up (single-, dual-chamber, CRT-D)
- Number of routine in-clinic visits per year
- Mean duration of in-clinic visits (single-, dual-chamber, CRT-D)
- Number and type of health care personnel involved in in-clinic visits
- Proportion of visits with ICD reprogramming
- Clinical evaluation performed at the time of routine ICD follow-up
- Presence of a structured heart failure management program in the center

Adoption and routine use of RM:

- Number of ICD in remote follow-up (single-, dual-chamber, CRT-D)
- Number of routine in-clinic visits per year in RM patients
- Frequency of scheduled remote interrogations
- Number and type of health care personnel involved in remote visits

###  The Data

Continuous data are expressed as means (standard deviations) or medians and interquartile ranges. Categorical data are expressed as percentages. Differences between mean data were compared by means of a *t* test for Gaussian variables, and by the Mann-Whitney nonparametric test for nonGaussian variables. Differences in proportions were compared by means of chi-square analysis or Fisher’s exact test, as appropriate. A *P* value <.05 was considered significant for all tests. All statistical analyses were performed by means of STATISTICA software, version 7.1 (StatSoft, Inc, Tulsa, OK, USA).

## Results

### Routine ICD Follow-Up

**Figure 2:**
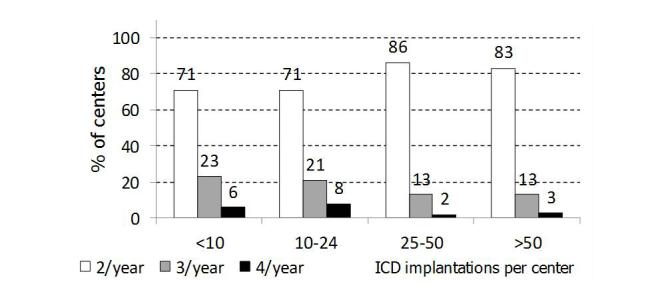
Frequency of scheduled in-clinic follow-up visits in relation to ICD implantation volume.

The frequency of routine in-clinic visits was 2 per year in 158/206 (76.7%) centers, 3 per year in 37/206 (18.0%) centers and 4 per year in 10/206 (4.9%) centers. Figure 2 shows the frequency of scheduled visits in relation to ICD implantation volumes; only a trend toward less frequent visits was seen in high-volume centers (*P*=.07).

Follow-up examinations were performed by a cardiologist in 203/206 (98.5%) centers and by more than one health care worker in 184/206 (89.3%). In 133/206 (64.6%) survey centers, the patient’s clinical status was not assessed during routine in-clinic follow-up, these visits being devoted exclusively to checking the ICD. In 75/206 (36.4%) centers, ICD patients were included in structured heart failure management programs. The reported duration of in-clinic follow-up visits was 15 (SD 7) minutes for single-chamber ICD, 16 (SD 8) minutes for dual-chamber ICD, and 20 (SD 9) minutes for cardiac resynchronization therapy ICD (CRT-D). Device reprogramming was required in (10%) (25th-75th percentile: 7-20) of visits.

### Adoption and Routine Use of RM

**Figure 3:**
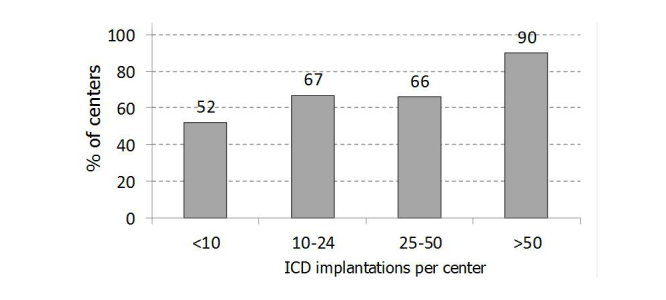
Current RM utilization, stratified by ICD implantation volume.

There were 137/206 (66.5%) responding centers that had already adopted an RM system for remote ICD interrogation. Figure 3 shows the proportions of centers using RM, stratified by ICD implantation volume.

In centers currently using RM systems, the proportion of ICD patients remotely monitored was 15% (25th-75th percentile: 5-30) for single-chamber ICD, 15% (25th-75th percentile: 5-35) for dual-chamber ICD, and 20% (25th-75th percentile: 10-42) for CRT-D. Remote ICD interrogations were scheduled every 3 months (25th-75th percentile: 1-3), and were performed by a cardiologist in 124/137 (90.5%) centers (*P*<.001 versus in-clinic visits). After the adoption of RM, the mean time between visits increased from 5 (SD 1) to 8 (SD 3) months (*P*<.001). Specifically, the frequency of in-clinic visits was decreased in 41/105 (39.0%) of centers routinely performing 2 visits per year in nonRM patients, in 15/24 (62.5%) of centers performing 3 visits per year, and 5/7 (71.4%) of centers performing 4 visits per year (*P*=.04).

## Discussion

### Results of the Survey

The results of the Italia-RM survey confirm that, in current clinical practice, in-clinic ICD follow-up visits consume a large amount of health care resources. Internet-based RM is becoming a new standard for the follow-up of patients with active implantable cardiac devices [13]. Nonetheless, the results of this survey show that it is not extensively used in Italy. Although the majority of Italian centers have begun to implement RM in their clinical practice, most of their patients continue to be followed-up by means of routine in-clinic visits.

A joint European Heart Rhythm Association (EHRA)-Eucomed survey [2] conducted in centers in seven European countries indicated that, in “real-world” clinical practice, resource utilization related to the follow-up of implantable cardiac devices places a considerable burden on arrhythmia services. The survey revealed that most follow-up examinations involved two staff members (usually a cardiologist and a nurse), and that visits lasted about 20 minutes. The authors hypothesized that most arrhythmia services may be reaching, or have already reached, their maximum workload capacity; this is in agreement with the findings of a previous survey conducted by the Heart Rhythm Society, which showed that follow-up examinations were the most frequent activities performed by electrophysiologists [1].

### Clinical Evaluations

In agreement with these findings, we ascertained that 184/206 (89.3%) of follow-up visits involved two staff members, and that a cardiologist attended 203/206 (98.5%) of examinations. By contrast, in the majority of survey centers, clinical evaluation by physicians was not performed at the time of routine device follow-up, although recommendations suggest that the clinical status of the patient should be reviewed during follow-up, as it may influence subsequent management [14]. Clinical evaluations may not be performed owing to the lack of time and resources, or may be carried out separately from the device check by physicians in charge of the clinical management of the patient. However, only a minority of responding centers reported including ICD patients in structured heart failure management programs.

Interestingly, it was recently demonstrated that patients who did not undergo clinical examination during device follow-up visits had a better attitude towards RM and were more appreciative of its timesaving advantage [15]. Thus, it was suggested that the stimulus to experience new device-check modalities could lie substantially in perceiving the current modalities as unsatisfactory. Anyhow, published reports on preliminary experiences of RM have consistently shown a high level of patient acceptance and satisfaction with RM [16,17]. Therefore, the limited adoption in current clinical practice should not be ascribed to a lack of acceptance by patients.

### RM Usage in European Clinical Practice

In 2010, an EHRA survey measured the use of RM in 61 European centers in 15 countries [10]. The authors reported that 52/61 (85%) of the centers already had experience of RM systems, and that management of the data collected in the majority of these centers was delegated to a dedicated allied professional. In 2011, a second survey, performed in 40 EHRA centers, showed less encouraging results [11]; RM was reportedly used routinely in CRT-D and ICD patients by only half of the centers.

The Italia-RM nation-wide survey analyzed the practice of ICD follow-up and the current use of RM systems in a large number of implanting centers in Italy. The participating centers represent about half of the Italian implanting centers and performed 5534/8998 (61.50%) of all ICD implantation procedures in 2011. Moreover, the participating centers displayed wide variability in the volume of procedures and were well distributed throughout the country.

Our analysis showed that RM systems have so far been adopted by 137/206 (66.5%) of centers for remote ICD interrogation. In each center, the median proportion of patients remotely monitored ranged from 15% of single-chamber ICD to 20% of CRT-D. The more frequent use of RM in CRT-D may be explained by the need to monitor sicker patients with greater continuity. Nonetheless, a recent analysis of the actions taken during in-clinic follow-up examinations suggested that the lower incidence of visits eliciting clinical or device-related action in the single- or dual-chamber ICD population should encourage the use of RM in these patients [18].

### Device Reprogramming

In our survey, device reprogramming was reported to be necessary in 10% of visits. Similarly, Mascioli et al [18] reported that device reprogramming was performed in 12% of scheduled visits. Boriani et al [2] reported a higher proportion of device reprogramming (about 30%) and a significant impact of reprogramming on the duration of the visit. However, it has been demonstrated that, following an initial optimization period, the frequency of device reprogramming declines and RM systems may become a more attractive alternative to in-clinic visits [19].

In general, RM may be timesaving for scheduled, nonactionable transmissions, while transmissions with clinically important findings and poor patient compliance have considerable workflow implications [20]. Therefore, in order to implement RM in standard clinical practice, new organizational models need to be developed in which nurses are responsible for training patients, entering and reviewing data, submitting critical cases to physicians, contacting patients, and ensuring patient compliance [21,22]. Recently, Ricci et al [23] reported that an outpatient clinic workflow model based on primary nursing could be extremely effective and could reduce resource consumption. Specifically, they showed that nurses could perform 76% of remote interrogation sessions. However, our results revealed that, in the vast majority of centers, remote ICD interrogations continued to be performed by a cardiologist.

### RM Visit Scheduling

In accordance with recommendations [14], routine in-clinic ICD examinations were performed every 3-6 months in our centers. However, it seems that high-volume centers tend to schedule visits less frequently, although this trend was nonsignificant. Similarly, the use of RM systems seems to be greater in high-volume centers. However, the main reason for adopting RM appears to be the prospect of improving the quality of care rather than reducing the workload in the centers. Indeed, remote transmissions were scheduled every 3 months; thus, the interval between ICD interrogations was reduced. Moreover, although the adoption of RM generally enabled the time between in-clinic visits to be increased, the majority of centers that scheduled less frequent visits prior to the adoption of RM were seen to have maintained the same number of in-clinic visits per year.

### Remote Interrogation of ICD Patients

The first reports on RM systems for the remote interrogation of ICD patients in Europe date back to around 10 years ago. Nonetheless, the results of the present survey show that it has only been partially adopted in Italy and that the majority of ICD patients continue to be followed-up by means of routine in-clinic visits.

Ostensibly, RM is more attractive for high-volume centers, where arrhythmia services may be overcrowded. Moreover, within each center, RM may be preferentially allocated to patients undergoing *de novo* ICD implantation, patients who are more compliant, or those to whom standard in-clinic visits cause greater inconvenience.

Appropriate reimbursement by health care systems and insurance companies, which is currently lacking in Italy and other European countries, is critical to stimulating the widespread adoption of RM [24]. Similarly, the adoption of new organizational models in the centers is warranted, in order to effectively and efficiently implement RM in standard clinical practice, converting this innovative approach to a cost-saving solution for patients, hospitals, and the public payer [23,25].

### Conclusions

In conclusion, in-clinic ICD follow-up visits currently consume a large amount of health care resources. Internet-based RM has been developed as a cost-effective solution for the management of patients with implantable cardiac devices. However, we showed that RM has only partially been adopted in Italy and, although many centers have begun to implement RM in their clinical practice, the majority of their patients continue to be routinely followed up by means of in-clinic visits.

## Multimedia Appendix 1

A complete list of participating centers.

## Abbreviations

AIAC: Italian Society of Arrhythmology and Pacing
CRT-D: cardiac resynchronization therapy ICD
EHRA: European Heart Rhythm Association
ICD: implantable cardioverter defibrillator
RM: remote monitoring
