# Supplementary material for: Remote Monitoring for Implantable Defibrillators: A Nationwide Survey in Italy
Source: Interact J Med Res. 2013 Sep 20;2(2):e27. doi: 10.2196/ijmr.2824 (PMC3786126; doi:10.2196/ijmr.2824)
Supplement: Supplementary file 1 [file ijmr_v2i2e27_app1.pdf]

ONLINE APPENDIX

Centers participating in the Italia-RM survey are listed below.

Villa dei fiori (Acerra): Paolo Gallo; Ospedale (Acqui Terme): Antonio Visconti; ASO Alessandria (Alessandria): Maura Giglio; Ospedale Umberto 2 (Altamura): Rodio Giovanna; AOU Ospedali Riuniti (Lancisi, Salesi, Umberto I) (Ancona): Mario Luzi; P.O. Bonomo (Andria): Francesco Bartolomucci, Nicola Di Tacchio, Giovanni Saggese; Ospedale Civile di Anzio (Anzio): Giuliano Pavoni; Ospedale S. Donato (Arezzo): Alessandro Fabiani; Sant'Ottone Frangipane (Ariano Irpino): Giuseppe Bianchino, Gianvito Manganelli,; O C Arzignano -Cazzavillan (Arzignano): Cosimo Perrone; Ospedale Mazzoni (Ascoli Piceno): Renato Marconi; Ospedale civile di Asti cardiologia (Asti): Paolo Di Donna; AO S.G. Moscati (Avellino): Ferdinando Alfano; PO G. Moscati (Aversa): Domenico Sarno; Ospedale San Paolo (Bari): Felice Giusti; C.D.C. Villa Bianca (Bari): Cosimo Di Candia; C.d.C. Santa Maria (Bari): Massimiliano Faustino; ASL BA Osp. "Di Venere" (Bari): Massimo Vincenzo Bonfantino, Carlo D'Agostino , Michele Palella; Cardiologia Universitaria Policlinico Bari (Bari): Stefano Favale , Massimo Iacoviello; OU Cardiologia ospedaliera Policlinico Bari (Bari): Domenico Carretta, Giuseppe Santoro; Ospedale Monsignor A.R. Di Miccoli Barletta (Barletta): Angelo Raffaele Mascolo, Michele Russo; Ospedale di Bassano del Grappa (Bassano del Grappa): Mirco Zadro; FateBeneFratelli (Benevento): Michele Della Porta; A.O. g. Rummo - Cardiologia interventistica (Benevento): Antonio Polcino; Istituto Humanitas Gavazzeni (Bergamo): Giosuè Mascioli; Ospedale Papa Giovanni XXII (Bergamo): Paolo De Filippo; Ospedale degli Infermi (Biella): Biondino Marenna; Ospedale Civile (Bisceglie): Vincenzo Massari; Bologna Ospedale Maggiore (Bologna): Gaetano Barbato, Valeria Carinci, Francesco Pergolini; Ospedale di Bolzano (Bolzano): Werner Rauhe; Borgo San Lorenzo (Borgo San Lorenzo): Domenico Rossi; Ospedale S. Pietro e Paolo (Borgosesia): Vincenzo Magnano; Ospedale SS. Trinità di Borgomanero (Borgomanero): Paola Paffoni;

Fondazione Poliambulanza (Brescia): Domenico Pecora; Istituto Clinico S. Anna (Brescia): Armando Gardini; Ospedale Perrino (Brindisi): Raffaele Rollo; A.O. Brotzu (Cagliari): Achille Giardina; PO SS. Trinità (Cagliari): Antonella Sanna; P.O. Gravina e Santo Pietro (Caltagirone): Giacomo Chiarandà; Fondazione di ricerca e cura Giovanni Paolo II (Campobasso): Quintino Parisi, Matteo Santamaria; Ospedale San Pietro Cosma (Camposampiero): Pietro Turrini, Roberto Verlato; Ospedale Ferrari (Casarano): Donato Melissano; Ospedale Santo Spirito (Casale Monferrato): Gabriele Dell'Era, Fabrizio Pizzetti; A.O. sant'Anna e San Sebastiano (Caserta): Miguel Viscusi; Ospedale San Giacomo USL 8 (Castelfranco Veneto): Fabia Merlini, Nicola Pellizzari, Gino Valente; Ospedale San Leonardo (Castellamare di Stabia): Ciro Guastaferro, Giovanni Russo; P.O. Ferrari (Castrovillari): Giovanni Bisignani, Silvana De Bonis; ARNAS Garibaldi Centro (Catania): Giuseppe Doria; A.O. Pugliese Ciaccio (Catanzaro): Giampiero Maglia; PO San Pellegrino (Catiglione delle Stiviere): Roberto Brunelli; P.O. Tatarella (Cerignola): Michele Cannone, Vincenzo Raddato; SS Annunziata (Chieti): Enrico Di Girolamo; Ospedale di Chioggia (Chioggia): Gabriele Boscolo; Ospedale Civio di Chivasso (Chivasso): Giuditta Corgnati; Ospedale (Ciriè): Claudia Amellone; P.O. Città di Castello (Città di Castello): Federico Fedeli; Ospedale S. Paolo (Civitavecchia): Sergio Calcagno; Ospedale Parodi Delfino (Colleferro): Salvatore Toscano; Ospedale di Conegliano (Conegliano): Giuseppe Allocca, Elena Marras, Nadir Sitta; A.O. Annunziata- Ospedale Civile di Cosenza (Cosenza): Antonello Talarico; A.O. "San Giovanni di Dio"- Crotone (Crotone): Massimo Elia; Santa Croce e Carle (Cuneo): Antonello Vado; A.O. Desenzano del Garda (Desenzano del Garda): GianPaolo Gelmini, Tommaso Bignotti; Ospedale S. Biagio (Domodossola): Antonio Mazzuero; S. M. Addolorata Dell'Olmo (Eboli): Angelo Carbone, Angelo Catalano; S. Giuseppe (Empoli): Attilio Del Rosso; Esine Vallecamonica (Esine): Antonio Farinelli; Cardiologia Ospedale Civile (Este): Barbara Ignatiuk; Az. Ospedaliera ORMN - PO di Fano (Fano): Andrea Pozzolini; Area Vasta N. 4 Ospedale di Fermo (Fermo): Paolo Paoloni; Arcispedale Sant'Anna (Ferrara): Matteo Bertini,

Tiziano Toselli; Ospedale Santa Maria Nuova (Firenze): Sergio Cerisano; Ospedale Riuniti Foggia, Scuola Universitaria di Cardiologia (Foggia): Matteo Di Biase , Pierluigi Pellegrino; Ospedale San Giovanni Battista (Foligno): Gianluca Savarese; Ospedale Morgagni (Forlì): Alberto Baldini, Paolo Golia; PO Dario Camberlingo (Francavilla): Aurelio Tarentini; Ospedale Fabrizio Spaziani (Frosinone): Luigi Carbonardi; Opsedale Sacro Cuore - Gallipoli (Gallipoli): Walter Colazzo; P.O Gavardo (A.O. Desenzano del Garda) (Gavardo): Marco Racheli; Ambulatorio Cardiologico Ospedale ASL 3 (Genova): Sergio Setti; Az. Osp. San Martino - Parte ospedaliera (Genova): Paolo Rossi; Ospedale Villa Scassi ASL3 (Genova Sampierdarena): Alessanro Mocini; Ospedale Civile di Gorizia (Gorizia): Luca Perazza; Ospedale Gubbio - Gualdo Tadino (Gubbio): Marco Ridarelli; Ospedale di Imperia (Imperia): Roberto Mureddu; Ospedale (Isernia): Bruno Castaldi; I.N.R.C.A. (Istituto Nazionale Riposo e Cura per Anziani) (Ancona): Marinella Marini; ASL TO 4 (Ivrea): Paolo Pistelli; Ospedale S. Andrea (La Spezia): Sandra Badolati; San Salvatore Coppito (L'Aquila): Raffaele Luise; Ospedale S. M. Goretti (Latina): Damiano Coletta; Ospedale Manzoni (Lecco): Franco Ruffa; UOC Cardiologia Ospedale Vito Fazzi (Lecce): Ennio Pisanò; Ospedale civile (Legnano): Matteo Mariani; AUSS21 "Matersalus" Legnago UO Cardiologia (Legnago (VR)): Giorgio Morando, Paola Guarise, Gabriele Zanotto; Ospedale Versilia (Lido di Camaiore): Alessio Lilli; ospedale Maggiore (Lodi): Fabio Lissoni; Ospedale Campo di Marte (Lucca): Davide Giorgi; Clinica San michele (Maddaloni): Antonio De Simone, Vincenzo La Rocca; Ospedale Fornaroli (Magenta): Roberto Turato; Ospedale di Manerbio (A.O. Desenzano del Garda) (Manerbio): Carlo Ferretti; Carlo Poma (Mantova): Patrizia Pepi; P.O. Paolo Borsellino (Marsala): Calogero Puntrello; Ospedale di Massa (Massa): Giuseppe Arena; Ospedale Madonna delle Grazie (Matera): Giancarlo Calculli; Ospedale F. Tappeiner cardiologia (Merano (BZ)): Rupert Paulmichl; C.D.C. Montevergine (Mercogliano): Francesco Solimene; A.O. Papardo Piemonte (Messina): Santina Patanè; Ospedale dell'Angelo (Mestre): Gianni Gasparini; Istituto Clinico Sant'Ambrogio (Milano): Stefania Panigada; P.O. Fogliani. Milazzo (Milazzo):

Giovanni Pizzimenti, Ludovico Vasques; Ospedale civile Mirano (Mirano): Emanuele Bertaglia; Policlinico di Modena (Modena): Edoardo Casali; Ospedale di Baggiovara (Modena): Sabbatani Paolo, Mauro Zennaro; Ospedale Santa Croce (Moncalieri): Catia Checchinato; U.O. Cardiologia UTIC Osp. S. Giacomo (Monopoli): Giangiuseppe Dalena; Ospedale S. Valentino (Montebelluna): Gianfilippo Neri; P.O. di Montepulciano (Montepulciano): Umberto Giannotti; Ospedale la Gruccia - Valdarno (Montevarchi): Francesco De Tommasi; A.O. A. Cardarelli (Napoli): Ruocco Antonio; AOU Federico II (Napoli): Maurizio Santomauro; Monaldi - Elettrostimolazione (Napoli): Vincenzo Caprioli; Clinica Mediterranea (Napoli): Assunta Iuliano, Giuseppe Stabile; Fatebenefratelli di Napoli Buonconsiglio (Napoli): Emilio Attena, Raffele Sangiuolo; Ospedale dei Colli, Plesso Monaldi (Napoli): Valter Bianchi, Ciro Cavallaro, Antonio D'Onofrio; Ospedale Loreto Mare (Napoli): Michelagelo Canciello , Raimondo Calvanese, Bernardino Tuccillo; Ospedale S.G.Bosco (Napoli): Gregorio Covino, Mario Volpicelli; Ospedali dei PellegrinI (Napoli): Valentino Ducceschi, Vincenzo Tavoletta; Policlinico Federico II (Napoli): Vincenzo Liguori, Antonio Rapacciuolo; Cardiologia Sacro Cuore (Negrar): Giulio Molon; Umberto I (Nocera): Nicola Capuano; AOU maggiore della Carità (Novara): Eraldo Occhetta; Ospedale san Giacomo (Novi Ligure): Gabriele Zaccone; Ospedale S.Francesco (Nuoro): Francesca Amadori; P.O. Giovanni Paolo II (Olbia): Isabella Simongini; Clinica S. rocco di Franciacorta (Ome): Armando Gardini; San Luigi Gonzaga (Orbassano): Giampaolo Varalda; P.O. San Martino (Oristano): Francesco Dettori; Ospedale G.B. Grassi (Ostia Lido): Roberto Colaceci; Ostuni (Ostuni): Elio Spennati; Clinica Cardiologica (Padova): Franco Antonio Folino; Centro cardiologico De Martino (Pagani): Umberto De Martino; Casa di Cura Maria Eleonora Hospital (Palermo): Ernesto Lombardo; A.O. Univ.Policlinico Paolo Giaccone (Palermo): Gianfranco Ciaramitaro; A.O. Osp.li Riuniti "Villa Sofia - Cervello" - PO Villa Sofia (Palermo): Orazio Pensabene; P.O. Barone Romeo (Patti): Antonino Lo Cascio; A.O. Ospedali riuniti Marche Nord P.O. Pesaro (Pesaro): Attilio Pierantozzi; Ospedale civile G. da Saliceto (Piacenza): Luca Rossi, Guido

Rusticali, Giovanni Villani; Ospedale Santa Corona (Pietra ligure): Daniela Sanfelici; Destra secchia (Pieve di Coriamo): Daniela Pozzetti; Pinerolo (Pinerolo): Riccardo Riccardi; AO Pisana Ospedale Cisanello (Pisa): Adriano Boem; Osp. Civile Giovanni Paolo II (Policoro): Andrea Andriani; Policlinico San Pietro (Ponte San Pietro): Antoine Kheir; Ospedale Felice Lotti (Pontedera): Stefano Giaconi, Alessandra Menegato; Santa maria delle Grazie (Pozzuoli): Mattia Liccardo; Ospedale di Vaio - Fidenza (PR) (Fidenza): Stefano Baccarini; Ospedale Misericordia e Dolce (Prato): Tiziana Giovannini; Santa Maria Degli Angeli (Putignano): Giovanni Ferraro; Ospedale di Ravenna (Ravenna): Massimo Margheri, Maurizio Piancastelli; Arcispedale Santa Maria Nuova (Reggio Emilia): Matteo Iori, Fabio Quartieri; Ospedale di Stato (Rep. Di San marino): Liano Marinelli; Ospedale Degli Infermi (Rivoli Torino): Anna Ferraro; Policlinico Casilino (Roma): Leonardo Calò; Association Columbus (Roma): Gianluca Comerci; Policlinico Umberto I (Roma): Giuseppe Giunta; Ospedale Sant'Eugenio (Roma): Filippo Lamberti; Ospedale San Pietro FateBeneFratelli (Roma): Daniele Porcelli; Ospedale di Rovigo (Rovigo): Francesco Zanon; Casa Sollievo della Sofferenza (S. Giovanni Rotondo): Raimondo Massaro, Domenico Potenza; Clinica Tortorella (Salerno): Natale Marrazzo; A.O. Ruggi D'Aragona e S. Giovanni di Dio (Salerno): Andrea Campana; Casa di cura Villa del Sole (Salerno): Francesco Solimene, Giuseppe Stabile; Ospedale D. Fracastoro USLL 20 Verona (San Bonifacio): Andrea Zorzi; ULSS 4 Alto Vicentino (Santorso Schio): Sergio Cannas; Ospedale di saronno (Saronno): Stefano Rossi; Nuovo osedale Civile (Sassuolo): Francesco Melandri; Ospedale Scorrano ASL Lecce (Scorrano): Leo Marsano; Monaldi (Seconda Univerisità Napoli) (Napoli): Ernesto Ammendola , Giuseppe Pacileo, Lucio Santengelo; Ospedale Bolognini (Seriate): Vittorio Giudici, Alessandro Locatelli; AOU Siena (Siena): Claudia Baiocchi; SOC Cardiologia AOVV sondrio (Sondrio): Maurizio Moizi; Ospedale SS. Trinità (Sora): Francesca Alfonsi, Alberto Scaccia; Ospedali Riuniti Area Penisola Sorrentina (Sorrento): Costantino Astarita, Prospero Stella; ASP di Messina- P.O. "San Vincenzo"- Taormina (Taormina): Giuseppe Calvagna; CDC Villa Verde (Taranto): Concetto La

Rosa; Ospeale Moscati - ASL Taranto (Taranto): Patrizio Gatto; Ospedale Mazzini (Teramo): Paolo Serra; Villa Maria Pia (Torino): Bruno Pezzulich; Sangiovanni Battista "Molinette" Cardio OSP (Torino): Carlo Budano; San Giovanni Battista "Molinette " Cardio UNIV (Torino): Piergiorgio Golzio; Ospedale Santa Chiara (Trento): Lorena Gramegna; UO Cardiologia AO G. Panico (Tricase): Pietro Palmisano; Polo Cardiologico Cattinara (Trieste): Luca Salvatore; Ospedale Santa Maria della Misericordia (Udine): Domenico Facchin, Alessandro Proclemer, Luca Rebellato; Ospedale Varese fondazione Macchi (Varese): Fabrizio Caravati; Ospedale Civile P. Colombo (Velletri): Natale Di Belardino; Ospedale civile di Venezia, SS Giovanni e Paolo (Venezia): Alessandro Vaglio; Cardiologia ASL Verbania (Verbania): Gabriele Iraghi; Ospedale S. Andrea (Vercelli): Lorella Barbonaglia; U.O. Cardiologia - A.O. Verona Borgo Trento (Verona): Ruggero Tomei; Ospedale Civile - Vicenza (Vicenza): Carlo Bonanno; Ospedale Belcolle (Viterbo): Massimiliano Campoli, Mario Malavasi, Massimo Sassara.
